# Dietary Green Alfalfa Supplementation Reduces Backfat Thickness and Improves Muscle Water-Holding Capacity in Diqing Tibetan Pigs

**DOI:** 10.3390/foods15142528

**Published:** 2026-07-17

**Authors:** Hete Huang, Xinpeng Li, Kang Zhang, Bingkun Liang, Siya Bai, Xinxing Dong, Dawei Yan

**Affiliations:** College of Animal Science and Technology, Yunnan Agricultural University, Kunming 650201, China; huanghete2020@163.com (H.H.); lixinpeng9812@163.com (X.L.); 13732717494@163.com (K.Z.); 15118124@njau.edu.cn (B.L.); 19988155290@163.com (S.B.)

**Keywords:** Diqing Tibetan pig, green alfalfa, multi-omics combination, muscle quality

## Abstract

Feed scarcity constrains livestock production, particularly on the Qinghai-Tibet Plateau. The effects of green alfalfa (GA) on Diqing Tibetan pig performance remain unclear. This study aimed to evaluate GA effects on Diqing Tibetan pig performance and to explore the potential underlying mechanisms through integrated metagenomic, transcriptomic, and metabolomic analyses. Thirty-six Diqing Tibetan pigs were randomly assigned to two groups and fed either a basal diet or a diet containing 90% basal diet and 10% GA. GA did not adversely affect growth performance but reduced 6–7 rib backfat thickness and muscle water loss rate by 19.79% (FDR = 0.027) and 17.80% (FDR = 0.036), while increasing muscle moisture content by 3.51% (FDR = 0.036). GA increased cecal microbial alpha diversity, Bacteroidota-related taxa, and functional genes related to lipid and vitamin metabolism, while decreasing Bacillota and *Lactobacillus johnsonii*. In the longissimus dorsi, *TNNI1*, *MYL2* and *MYL3* were upregulated, whereas *FOS* and *FOSB* were downregulated; GA increased vanillyl alcohol, L-histidine, LPE (0:0/22:5), and licochalcone B, but decreased glyceryl monostearate, benzaldehyde, cortisol, tryptamine, 4-ethyloctanoic acid, 8-methylnonanoic acid, and purine. Overall, 10% GA reshaped gut microbial, muscle transcriptomic, metabolomic profiles and collectively influenced 6–7 rib backfat thickness and muscle water-holding capacity in Diqing Tibetan pigs.

## 1. Introduction

Pork accounts for approximately 30% of global meat consumption and provides humans with high-quality protein, B vitamins, zinc, iron, and other essential nutrients [1]. As consumers increasingly value the health attributes, eating quality, and production methods of pork, improving pork quality while maintaining production efficiency has become an important goal for the pig industry [2]. Meanwhile, the shortage of feed resources is one of the major challenges currently facing pig production [3]. Livestock and poultry can convert unconventional feed resources that are not edible by humans into animal products, which can alleviate competition between humans and livestock for grain [4], reduce feed costs, and improve production efficiency [5].

Alfalfa is known as the “king of forages” and is rich in protein, dietary fiber, vitamins, minerals, and bioactive molecules such as saponins, polysaccharides, and flavonoids [6]. Increasing the dietary inclusion of alfalfa meal from 20% to 30% in Heigai pigs significantly reduced backfat thickness and increased the contents of flavor amino acids and essential amino acids [7]. In Duroc × Landrace × Yorkshire (DLY) pigs, dietary supplementation with 25% alfalfa leaf meal significantly increased the intramuscular fat content of the longissimus dorsi (LD) and significantly upregulated the expression of genes involved in lipid transport (*CD*36 and *FATP*4) and lipid synthesis (*ACCα*, *PGC*1*α*, *PPARα*, *PPARδ*, and *SREBP*1-*c*) [8]. In DLY pigs, the inclusion of 10% alfalfa silage improved muscle marbling and water-holding capacity, reduced drip loss, reshaped the gut microbiota, and altered short-chain fatty acid levels [9]. Alfalfa can be processed into hay, silage, meal, pellets, and other products; however, nutrient losses can readily occur during processing and storage [10]. Direct feeding of fresh forage to livestock and poultry may be a strategy for producing premium-quality meat [11]. Feeding green alfalfa directly can reduce nutrient losses [9], but few studies have reported the effects of direct feeding of green alfalfa on pig performance.

The Diqing Tibetan pig is a type of Tibetan pig and is distributed in the high-altitude regions above 3000 m in Diqing Tibetan Autonomous Prefecture, Yunnan Province [12]. It is a plateau-type breed that is adapted to cold and hypoxic environments and is known for strong fat deposition and good meat quality [13]. Diqing Tibetan pigs can be classified into large- and small-bodied types. Adult large-bodied pigs can reach 70–150 kg, with an average daily gain of 200–250 g/day during the fattening period; small-bodied pigs generally weigh 45–55 kg, with an average daily gain of 100–120 g/day during fattening [14]. The average litter size is approximately 5.46 piglets, indicating low reproductive performance [15]. Diqing Tibetan pork has a high protein content and is rich in unsaturated fatty acids and essential amino acids beneficial to human health [16]. Tibetan pig farming is an important source of income for local Tibetan farmers [17]. However, because Diqing Tibetan Autonomous Prefecture is located on the southeastern edge of the Qinghai-Tibet Plateau, with cold, hypoxic conditions and limited feed resources, high production costs severely constrain the development of the Tibetan pig industry and income growth among local farmers [18]. Developing forage resources suitable for local conditions and exploring their application value in growing-finishing pigs are therefore important for reducing production costs, increasing the added value of local pig industries, and promoting the sustainable development of animal husbandry in plateau regions [3,4,5].

Based on this background, we hypothesized that green alfalfa may improve meat quality by regulating lipid metabolism without reducing growth performance in Diqing Tibetan pigs. To test this hypothesis, green alfalfa was added to the diets of Diqing Tibetan pigs. Growth performance and carcass traits were used as primary outcomes, and meat quality and muscle chemical composition were used as secondary outcomes. Metagenomic, muscle transcriptomic, muscle metabolomic, and phenotypic data were integrated to explore potential mechanisms by which green alfalfa affects fat deposition and meat quality in Diqing Tibetan pigs. The findings provide a scientific basis for using green alfalfa in finishing pig production and offer a new perspective for mitigating competition between humans and livestock for grain and producing high-quality pork.

## 2. Materials and Methods

### 2.1. Ethical Statement

This experiment was approved by the Animal Ethics Committee of Yunnan Agricultural University (approval no. APYNAU202401001).

### 2.2. Nutritional Analysis of Green Alfalfa

One green alfalfa sample was collected at each of the early, middle, and late stages of the feeding trial. The nutrient composition of the experimental green alfalfa (*Medicago sativa*; fall dormancy rating, 6.0) was determined according to the relevant Chinese National Standards (GB/T). Moisture content was measured according to Determination of Moisture in Feeds (GB/T 6435); crude protein content was measured according to Determination of Crude Protein in Feeds, Kjeldahl Method (GB/T 6432); crude fat content was measured according to Determination of Crude Fat in Feeds (GB/T 6433); crude fiber content was measured according to Determination of Crude Fiber in Feeds (GB/T 6434); calcium content was measured according to Determination of Calcium in Feeds (GB/T 6436); and phosphorus content was measured according to Determination of Total Phosphorus in Feeds, Spectrophotometric Method (GB/T 6437). Gross energy and digestible energy for growing pigs were obtained from the nutritional values reported for the fresh aerial parts of green alfalfa in Feedipedia [19].

### 2.3. Feeding of Experimental Pigs

Thirty-six 4-month-old large-bodied Diqing Tibetan pigs of the same parity and similar body weight (equal numbers of males and females; all surgically sterilized, with the testes removed in males and the ovaries and uterus removed in females) were randomly assigned to two treatment groups, with three replicates per treatment and six pigs per replicate. The pigs were fed either a basal diet (CON) or a diet comprising 90% basal diet and 10% green alfalfa (GA) (Table 1). The inclusion ratios of the basal diet and green alfalfa were calculated on a dry matter basis. Previous intervention studies using different alfalfa meal inclusion levels (0%, 5%, 10%, and 15%) in DLY pigs showed that 10% had the best effect on butyrate production in the cecum and colon [20]. Both 10% alfalfa silage and 10% alfalfa meal have been shown to reshape the gut microbiota, alter short-chain fatty acid levels, and improve water-holding capacity and marbling scores [9]. Therefore, 10% green alfalfa was selected for dietary supplementation in the present study. All pigs were housed in the same pig building, had free access to water, and were fed three times daily (09:00, 13:00, and 19:00) with ad libitum access to feed. Feed offered and feed refusals were recorded daily by designated personnel. Each pig was weighed at the beginning and end of the trial to calculate average daily gain (ADG), total dry matter intake, and dry matter feed conversion ratio. The formulas were as follows: total dry matter intake = (basal diet × dry matter content) + (green alfalfa × dry matter content); dry matter feed conversion ratio = total dry matter intake/(final body weight − initial body weight); total digestible energy intake = total dry matter intake × dietary digestible energy. The experimental period lasted 135 days, from 2 August 2024 to 14 December 2024.

### 2.4. Sample Collection

At the end of the experiment, six pigs were randomly selected from each group (two pigs per replicate, including one male and one female). Each pig was individually electrically stunned, exsanguinated, dehaired, and had the hooves, tail, and viscera removed, while the leaf fat and kidneys were retained. The carcasses were then split along the midline. The longissimus dorsi (LD) muscle at the level of the last one to two thoracic vertebrae of the left carcass and cecal contents were collected. After removal of visible fat and fascia, the LD muscle was divided into two portions. One portion was immediately frozen in liquid nitrogen, transported to the laboratory, and stored at −80 °C for RNA-seq and metabolomic analyses. The other portion was stored at −20 °C, transported to the laboratory, and used for chemical composition and tenderness determination. Cecal contents were immediately snap-frozen in liquid nitrogen, transported to the laboratory, and stored at −80 °C for metagenomic analysis.

### 2.5. Determination of Carcass Traits and Meat Quality

#### 2.5.1. Determination of Carcass Traits

Carcass weight, backfat thickness, 6–7 rib backfat thickness, loin eye area, dressing percentage, lean meat percentage, fat percentage, bone percentage, and skin percentage were determined according to the Technical Specification for Determination of Carcass Traits in Lean-Type Pigs (Chinese Agricultural Industry Standard NY/T 825).

#### 2.5.2. Determination of Meat Quality

Samples were collected, and muscle pH, meat color, marbling, cooking loss, water loss rate, drip loss, and shear force were measured according to the Technical Procedures for Determination of Pork Quality (Chinese Agricultural Industry Standard NY/T 821).

### 2.6. Determination of Muscle Chemical Composition

After slaughter, the LD muscle from the left carcass was collected. Moisture content was determined according to the Chinese National Food Safety Standard: Determination of Moisture in Foods (GB 5009.3); crude protein content was determined according to the Chinese National Food Safety Standard: Determination of Protein in Foods (GB 5009.5); intramuscular fat content was determined according to the Chinese National Food Safety Standard: Determination of Fat in Foods (GB 5009.6); and crude ash content was determined according to the Chinese National Food Safety Standard: Determination of Ash in Foods (GB 5009.4).

### 2.7. Metagenomic Sequencing and Analysis of Cecal Contents

#### 2.7.1. DNA Extraction and Sequencing

##### Extraction and Quality Control of Total Microbial DNA

Total microbial DNA was extracted from cecal contents using the FastDNA Fecal Spin Kit (MP Biomedicals, Irvine, CA, USA). The integrity and concentration of the extracted DNA were assessed using 1.5% agarose gel electrophoresis and a NanoDrop 1000 spectrophotometer (Thermo Fisher Scientific, Waltham, MA, USA). DNA samples meeting the following criteria were stored at −20 °C until use: good integrity, DNA concentration ≥20 ng/μL, total DNA amount ≥1 μg, A260/A280 = 1.8–2.0, A260/A230 ≥ 1.8, and no obvious degradation on gel electrophoresis.

##### Library Construction and Sequencing

For each qualified sample, 1 μg of DNA was used for library preparation. Sequencing libraries were prepared using the Hieff NGS^®^ OnePot Pro DNA Library Prep Kit V4 one-step enzymatic DNA fragmentation library construction kit (Yeasen Biotechnology (Shanghai) Co., Ltd., Shanghai, China), and index codes were added to identify each sample. Briefly, DNA was randomly fragmented enzymatically to approximately 350 bp, followed by end repair, A-tailing, and ligation with full-length indexed sequencing adapters for Illumina sequencing and subsequent PCR amplification. PCR products were purified using the AMPure XP system (Beckman Coulter, Brea, CA, USA), and library size and distribution were analyzed using an Agilent 2100 Bioanalyzer (Agilent Technologies, Santa Clara, CA, USA). Metagenomic sequencing was performed on the Illumina NovaSeq 6000 platform (Illumina, San Diego, CA, USA) at a sequencing depth of 10 G.

#### 2.7.2. Alignment and Annotation of Raw Data

Raw data were first quality-filtered, adapter-trimmed, and length-filtered using fastp (v1.0.1) to obtain clean data. The clean data were aligned to the pig reference genome (Sscrofa11.1) using bowtie2 (v2.5.4). Host-derived reads aligned to the pig reference genome were removed using Samtools (v1.21), and reads that did not align to the pig reference genome were retained and aligned against the Kraken2 standard database (https://genome-idx.s3.amazonaws.com/kraken/k2_standard_20250714.tar.gz; accessed on 14 July 2025) (containing bacterial, archaeal, and viral reference sequences) using the Kraken2 package for annotation. Bracken was subsequently used to correct the abundance estimates of the Kraken2 annotation results. Taxonomic annotations were resolved to the species level, yielding classification and abundance matrices across seven taxonomic levels from kingdom to species.

#### 2.7.3. Microbial Taxonomic Analysis

Based on the microbial abundance matrix, the vegan package was used to calculate alpha-diversity indices, including observed taxa and the Shannon and Simpson indices. Beta diversity analysis was also performed using vegan, including principal coordinates analysis (PCoA) and non-metric multidimensional scaling (NMDS) based on Bray–Curtis distances. The microeco package was used to analyze microbial composition at the phylum, genus, and species levels and to assess between-group differences. LEfSe analysis was performed using microeco and vegan, and LDA bar plots were used to display differential taxa from the phylum to species levels.

#### 2.7.4. Functional Annotation of the Microbiota

The reads that did not align to the pig reference genome after Samtools filtering were assembled into contigs using MEGAHIT [21], and contigs shorter than 1000 bp were removed to improve the reliability of subsequent gene prediction and functional annotation. Genes were then predicted from the assembled contigs using MetaGeneMark. Predicted genes were dereplicated using CD-HIT, with genes showing ≥90% sequence similarity clustered into the same group to reduce redundancy caused by highly similar or duplicate gene sequences while preserving gene diversity as much as possible. DIAMOND was then used to align the sequences against the EggNOG database (http://EggNOG5.embl.de/download/emapperdb-5.0.2/; accessed on 17 July 2025) for functional annotation. Based on the functional pathway abundance matrix, the overall relative abundance of each functional pathway across all samples was calculated, and pathways with an overall relative abundance greater than 0.1% were subjected to between-group differential analysis to reduce the potential influence of extremely low-abundance functional terms on statistical comparisons.

### 2.8. Transcriptome Sequencing Analysis of the LD Muscle

#### 2.8.1. Transcriptome Sequencing

##### Extraction and Quality Control of Total RNA

The LD muscle was removed from the −80 °C freezer, and total RNA was extracted using TRIzol (Invitrogen, Carlsbad, CA, USA). RNA concentration and purity were then measured using a NanoDrop ND-1000 spectrophotometer (NanoDrop, Waltham, MA, USA), and RNA integrity was assessed using a Bioanalyzer 2100 (Agilent, Santa Clara, CA, USA) and agarose gel electrophoresis. RNA samples used for library construction were required to meet the following quality criteria: concentration > 50 ng/μL, RIN > 7.0, OD260/280 > 1.8, and total amount >1 μg, to ensure sufficient input for library construction, low protein or reagent contamination, and good integrity.

##### mRNA Enrichment and Fragmentation

mRNA with poly(A) tails was enriched using oligo(dT) magnetic beads (Thermo Fisher, Waltham, MA, USA). After the addition of a magnesium ion buffer, RNA was fragmented by high-temperature treatment (94 °C for 5–7 min).

##### Library Construction

Using fragmented RNA as the template, first-strand cDNA was synthesized with reverse transcriptase (SuperScript™ II, Invitrogen, Carlsbad, CA, USA). Second-strand cDNA was subsequently synthesized using *E. coli* DNA polymerase I and RNase H (NEB, Ipswich, MA, USA), with dUTP (Thermo Fisher, Waltham, MA, USA) incorporated for strand-specific library construction. Double-stranded cDNA was end-repaired to generate blunt ends, A-tailed at the 3′ ends, ligated to adapters with T overhangs, and then size-selected and purified using magnetic beads. The dUTP-containing second-strand cDNA was digested with UDG enzyme (NEB, USA, Ipswich, MA), followed by PCR amplification (95 °C for 3 min; 8 cycles of 98 °C for 15 s, 60 °C for 15 s, and 72 °C for 30 s; and 72 °C for 5 min) to generate libraries with an insert size of 300 ± 50 bp.

##### Sequencing

PE150 paired-end sequencing was performed on the Illumina NovaSeq 6000 platform (Illumina, San Diego, CA, USA) at a sequencing depth of 6 G.

#### 2.8.2. Transcriptome Data Analysis

Raw data were quality-controlled and filtered using fastp to obtain clean data. The clean data were aligned to the pig reference genome (Sscrofa11.1) using HISAT2 (v2.2.1). The SAM files generated from alignment were converted to BAM files using Samtools (v1.21), and featureCounts (v2.0.6) was then used to merge and process the BAM files into gene expression quantification files. TPM values were calculated from the gene expression quantification matrix to obtain a TPM matrix, and principal component analysis (PCA) was performed based on the gene expression quantification matrix. Differential expression analysis was performed using the R package DESeq2 with |log2(FoldChange)| > 1 and adjusted *p* value < 0.05 as screening criteria. The identified differentially expressed genes were subjected to GO and KEGG enrichment analyses using the DAVID website (https://davidbioinformatics.nih.gov/; accessed on 10 July 2025), and visualization was performed using the R package ggplot2. The differentially expressed genes were imported into the STRING website (https://cn.string-db.org/; accessed on 10 July 2025) for protein–protein interaction (PPI) analysis using *Sus scrofa* as the reference. The interaction score was set to 0.70 for high confidence, and hub genes were identified based on degree centrality. The constructed network was visualized and subjected to network topology analysis using Cytoscape software (v3.10.4).

#### 2.8.3. RT-qPCR Analysis

For RT-qPCR analysis, total RNA was independently reextracted from the same LD tissues used for RNA-seq using the RNA Simple Total RNA Kit (TIANGEN Biotech, Beijing, China), according to the manufacturer’s instructions. RNA concentration and purity were assessed using a NanoDrop spectrophotometer (Thermo Fisher Scientific, Wilmington, DE, USA), and RNA integrity was evaluated by agarose gel electrophoresis. Total RNA was subsequently reverse-transcribed into cDNA using the FastKing First-Strand cDNA Synthesis Kit (TIANGEN Biotech, Beijing, China) according to the manufacturer’s instructions. *GAPDH* was used as the internal reference gene. Target gene primers were designed using Primer Premier 5.0 and synthesized by Sangon Biotech (Shanghai) Co., Ltd. (Shanghai, China); primer sequences are listed in Table S1. Real-time quantitative PCR was performed using a SYBR Green qPCR kit (TIANGEN Biotech, Beijing, China; FP205) on an FQD-96A real-time fluorescence quantitative PCR instrument (Bioer, Hangzhou, China). The total PCR reaction volume was 20 μL, containing 10 μL of 2 × SuperReal PreMix Plus, 0.6 μL each of forward and reverse primers (10 μM), 1 μL of cDNA template, 0.5 μL of 50 × ROX Reference Dye, and 7.3 μL of RNase-free water. The amplification program was 95 °C for 15 min, followed by 40 cycles of 95 °C for 10 s and 60 °C for 20 s for annealing and extension. After amplification, melting curve analysis was performed from 60 to 95 °C to verify the specificity of the amplification products. Relative expression levels were calculated using the 2^−ΔΔCt^ method [14]. Welch’s independent-samples t-test was performed, and the results were visualized using R software (v4.3.3).

### 2.9. Widely Targeted Metabolomic Detection and Analysis

#### 2.9.1. Sample Preparation

The LD samples were removed from the −80 °C freezer and thawed on ice, then ground in liquid nitrogen. A 20 mg sample was placed in a centrifuge tube, and 400 μL of methanol-water extraction solution containing internal standards (methanol:water = 7:3, *v*/*v*; the composition, concentration, brand, and other information of the internal standards are provided in Table S2) was added. The mixture was vortexed at 2500 r/min for 5 min and left to stand for 15 min. Samples were centrifuged at 12,000 r/min for 10 min at 4 °C, and 300 μL of supernatant was collected and stored at −20 °C for 30 min. After centrifugation at 12,000 r/min for 3 min at 4 °C, the supernatant was collected for LC-MS analysis.

#### 2.9.2. Ultra-Performance Liquid Chromatography Conditions

The supernatant was divided into three equal aliquots and analyzed using three liquid chromatography methods. For positive ion mode, separation was performed on a T3 column (Waters ACQUITY UPLC HSS T3 C18, 1.8 μm, 2.1 mm × 100 mm; Waters Corporation, Milford, MA, USA). Mobile phase A was water containing 0.1% formic acid, and mobile phase B was acetonitrile containing 0.1% formic acid. The gradient elution program was as follows: 0–2 min, B increased from 5% to 20%; then to 60% within 3 min; then to 99% within 1 min and maintained for 1.5 min; followed by rapid return to 5% B within 0.1 min and equilibration for 2.4 min. Chromatographic conditions were as follows: column temperature, 40 °C; flow rate, 0.4 mL/min; injection volume, 2 μL or 5 μL. For negative ion mode using reversed-phase chromatography, the column and elution gradient were the same as those used in positive ion mode. For negative ion mode using HILIC, analysis was performed on a Waters ACQUITY UPLC BEH HILIC column (1 mm × 150 mm, 1.7 μm; Waters Corporation, Milford, MA, USA). Mobile phase A consisted of acetonitrile:water:methanol (80:10:10, *v*/*v*/*v*) containing 20 mM ammonium formate (pH 10.6), and mobile phase B consisted of acetonitrile:water (40:60, *v*/*v*) containing 20 mM ammonium formate. The elution gradient was as follows: 5% B increased to 20% B within 2 min; 20% B increased to 70% B from 2 to 3.5 min; 70% B increased to 95% B from 3.5 to 6.5 min and maintained for 1 min; followed by rapid return to the initial conditions.

#### 2.9.3. Mass Spectrometry and Acquisition Conditions for Non-Targeted and Widely Targeted Detection

The data acquisition system for non-targeted mass spectrometry comprised ultra-performance liquid chromatography (UPLC; ExionLC AD, https://sciex.com.cn/; accessed on 15 March 2025) and quadrupole-time-of-flight mass spectrometry (TripleTOF6600, AB SCIEX, Framingham, MA, USA). The data acquisition system for widely targeted detection comprised UPLC (ExionLC AD, https://sciex.com.cn/; accessed on 15 March 2025) and tandem mass spectrometry (MS/MS; QTRAP^®^, https://sciex.com/; accessed on 15 March 2025).

##### Quadrupole-Time-of-Flight Mass Spectrometry

Data acquisition was performed in information-dependent acquisition mode using Analyst TF 1.7.1 software. Ion source parameters were set as follows: ion source gas 1, 50 psi; ion source gas 2, 50 psi; curtain gas, 25 psi; temperature, 550 °C; declustering potential, 60 V and −60 V in positive and negative ion modes, respectively; and spray voltage, 5000 V and −4000 V in positive and negative ion modes, respectively. TOF MS full-scan parameters were set as follows: mass range, 50–1000 Da; accumulation time, 200 ms; dynamic background subtraction was enabled. Product ion scan parameters were set as follows: mass scan range, 25–1000 Da; accumulation time, 40 ms; collision energy, 30 V and −30 V in positive and negative ion modes, respectively; collision energy spread, 15; resolution, UNIT; charge state range, 1–1; ion intensity threshold, 100 cps. Isotope peaks within 4 Da were excluded; the mass tolerance was 50 ppm, and up to 18 candidate ions were monitored per cycle.

##### Tandem Mass Spectrometry

Analysis was performed on a QTRAP^®^ LC-MS/MS system equipped with an electrospray ionization turbo ion source. The system was operated in positive and negative ion modes and controlled by Analyst 1.6.3 software. ESI source operating parameters were as follows: ion source temperature, 500 °C; spray voltage, 5500 V and −4500 V in positive and negative modes, respectively; ion source gas I (GSI), ion source gas II (GSII), and curtain gas (CUR), 50, 50, and 25.0 psi, respectively; and collision gas (CAD), high. Instrument tuning and mass calibration were performed using 10 μmol/L and 100 μmol/L polypropylene glycol solutions in QQQ and LIT modes, respectively.

#### 2.9.4. Metabolite Identification and Quantification

Metabolites were qualitatively identified based on the Metware Database (MWDB), an in-house reference-standard database containing MS/MS spectra and retention time (RT) information; the DB-all public database integrated by Metware, including METLIN, HMDB, KEGG, and other databases; an AI-predicted library (version 4.0) constructed using the CFM-ID algorithm [22]; and MetDNA. For the MWDB/CFM-ID identification workflow, MWDB was used as the training set to construct a predicted spectral library using the CFM-ID model. Sample spectra were matched against database reference spectra from MWDB and the predicted spectral library using a cosine similarity algorithm. The matching criteria allowed mass errors of 25 ppm for precursor ions (Q1), 50 ppm for MS/MS ions, and 60 s for RT. The final MS/MS similarity score comprised fragment, forward-search, and reverse-search scores, with weights of 0.1, 0.3, and 0.6, respectively; metabolites with a final score of at least 0.3 were retained. Based on the multiple reaction monitoring (MRM) ion pairs, RTs, and other information for all identified metabolites, a targeted detection database was established. Metabolites in this database were then accurately quantified on a QTRAP-MS/MS platform using the multiple reaction monitoring mode of a triple quadrupole mass spectrometer.

#### 2.9.5. Screening of Differential Metabolites

PCA and orthogonal partial least squares-discriminant analysis (OPLS-DA) were used to evaluate overall metabolic differences among samples and the separation trend between groups. PCA was performed in R software (v4.1.2) using unit variance scaling. OPLS-DA was performed using the R package MetaboAnalystR (v1.0.1), and the data were log2-transformed and centered. The goodness of fit and cross-validated predictive ability of the OPLS-DA model were expressed as R^2^Y and Q^2^, respectively. To assess model robustness and the potential risk of overfitting, 200 permutation tests were used for internal validation. In each permutation, sample group labels were randomly shuffled and the OPLS-DA model was reconstructed; the R^2^Y and Q^2^ values of the permuted models were then compared with those of the original model. Variable importance in projection (VIP) values were obtained from the OPLS-DA model, and differential metabolites were screened using *p* < 0.05 and VIP > 1.0 as thresholds. The differential metabolites were annotated in the KEGG Compound database (http://www.kegg.jp/kegg/compound/; accessed on 20 March 2025), and the annotated metabolites were then mapped to the KEGG Pathway database (http://www.kegg.jp/kegg/pathway.html; accessed on 20 March 2025) for pathway analysis.

### 2.10. Integrated Multi-Omics Analysis

Association analyses were performed using relatively abundant taxa at the phylum, genus, and species levels, differentially abundant predicted functional pathways, hub genes in the PPI network, key metabolites, and meat quality traits. Pearson correlation analysis was used for the integrated analyses of cecal microbiota and predicted functional pathways, cecal microbiota and LD transcriptome, LD transcriptome and metabolome, and metabolome and meat quality. Correlation coefficients and two-sided *p* values were calculated. *p* values within each correlation matrix were adjusted for false discovery rate (FDR) using the Benjamini–Hochberg method, and 95% confidence intervals for correlation coefficients were calculated using 1000 bootstrap resampling iterations. FDR < 0.05 and |r| ≥ 0.70 were used as criteria for significant associations, and the 95% confidence intervals were used to evaluate the precision of correlation coefficient estimates.

### 2.11. Statistical Analysis

Growth performance, carcass traits, meat quality traits, and muscle chemical composition were analyzed using the pen as the statistical unit, and results are presented as means ± standard deviations. Statistical analyses were performed using IBM SPSS Statistics 27. Data normality was first assessed using the Shapiro–Wilk test, with *p* ≥ 0.05 indicating a normal distribution and *p* < 0.05 indicating a non-normal distribution. Normally distributed data were compared between two groups using Student’s t-test, whereas non-normally distributed data were analyzed using the Wilcoxon rank-sum test. The raw *p* values of the above phenotypic indicators were then adjusted for multiple testing using the Benjamini–Hochberg method, and the adjusted *p* values were used to evaluate statistical significance. Between-group differences in alpha diversity, phylum-, genus-, and species-level community composition, the Bacillota/Bacteroidota ratio, EggNOG-predicted functional pathways, peptide content among all identified metabolites, and peptide content among differential metabolites were assessed using the Wilcoxon rank-sum test. Between-group differences in metabolites were analyzed using Student’s t-test, whereas between-group differences in gene expression were tested using a negative binomial generalized linear model based on the Wald test. Beta diversity was visualized using PCoA and NMDS based on Bray–Curtis distance matrices, and significance was tested using PERMANOVA. Differential microbial biomarkers were identified using LEfSe analysis, in which the Kruskal–Wallis test was used to screen for differential microorganisms between groups and linear discriminant analysis scores were calculated to assess effect sizes.

## 3. Results

### 3.1. Nutritional Composition and Energy Values of Green Alfalfa

The nutrient composition of the green alfalfa used in this experiment is shown in Table 2. The moisture content of green alfalfa was 82.19%. On a dry matter basis, green alfalfa contained 13.73% crude protein, 4.19% crude fat, 26.26% crude fiber, 0.09% calcium, and 0.04% phosphorus. Its gross energy was 18.10 MJ/kg, and its digestible energy for growing pigs was 8.70 MJ/kg.

### 3.2. Dietary Green Alfalfa Supplementation Significantly Reduced 6–7 Rib Backfat Thickness in Diqing Tibetan Pigs

The growth performance results (Table 3) showed that final body weight, average daily gain, total dry matter intake, total digestible energy intake and dry matter feed conversion ratio did not differ significantly between the GA and CON groups. The carcass trait results (Table 4) showed that 6–7 rib backfat thickness was significantly reduced in the GA group, with a relative reduction of 19.79%.

**Table 3:** Effects of adding green alfalfa to the diet on the growth performance of Diqing Tibetan pigs.

Items	CON (*n* = 3)	GA (*n* = 3)	FDR
Initial Body Weight (kg)	18.48 ± 0.53	18.56 ± 0.80	0.936
Final Body Weight (kg)	86.61 ± 1.35	83.08 ± 1.80	0.238
Average Daily Gain (ADG) (g/day)	504.66 ± 12.11	477.95 ± 19.23	0.428
Total Basal Diet Intake (kg/head)	272.79 ± 23.90	236.85 ± 5.44	/
Total Green Alfalfa Intake (kg/head)	/	127.82 ± 3.28	/
Total Dry Matter Intake (kg/head)	235.96 ± 20.67	227.64 ± 5.20	0.688
Total Digestible Energy Intake (MJ/head)	2984.94 ± 261.47	2790.85 ± 63.76	0.650
Dry Matter Feed Conversion Ratio	3.50 ± 0.25	3.54 ± 0.06	0.889

Note: FDR values are *p* values adjusted using the Benjamini–Hochberg method. Differences between groups were considered statistically significant at FDR < 0.05. The same applies to Table 4 and Table 5.

**Table 4:** Effects of adding green alfalfa to the diet on the carcass characteristics of Diqing Tibetan pigs.

Items	CON (*n* = 3)	GA (*n* = 3)	FDR
Liveweight (kg)	89.08 ± 7.27	85.32 ± 2.22	0.688
Dressing Percentage (%)	69.53 ± 1.54	70.93 ± 1.00	0.650
Average Backfat Thickness (mm)	37.95 ± 6.13	34.43 ± 2.86	0.688
6–7 rib Backfat Thickness (mm)	50.98 ± 0.91	40.89 ± 1.93	0.027
Eye Muscle Area (cm^2^)	27.40 ± 3.90	28.55 ± 5.58	0.883
Lean Meat Percentage (%)	48.56 ± 4.85	50.71 ± 0.93	0.688
Fat Percentage (%)	32.02 ± 6.04	29.54 ± 1.62	0.688
Skin Percentage (%)	9.11 ± 0.55	8.66 ± 1.02	0.688
Bone Percentage (%)	10.31 ± 0.98	11.10 ± 0.53	0.650

**Table 5:** Effects of adding green alfalfa to the diet on meat quality and muscle chemical composition of Diqing Tibetan pigs.

Items	CON (*n* = 3)	GA (*n* = 3)	FDR
pH_45min_	6.32 ± 0.23	6.22 ± 0.12	0.688
pH_24h_	5.91 ± 0.22	5.79 ± 0.17	0.688
Meat color	3.83 ± 0.14	4.17 ± 0.29	0.600
Marbling	5.00 ± 1.15	4.50 ± 0.75	0.688
Cooking loss (%)	36.31 ± 1.31	36.78 ± 0.90	1.000
Rate of water loss (%)	19.05 ± 0.52	15.66 ± 0.75	0.036
Drip loss (%)	2.41 ± 0.38	2.17 ± 0.58	0.688
Shear force (N)	31.67 ± 1.85	33.64 ± 0.53	0.496
Moisture (%)	71.25 ± 0.53	73.75 ± 0.50	0.036
Crude protein (%)	22.65 ± 0.19	21.98 ± 0.20	0.081
Intramuscular fat (%)	4.23 ± 0.35	3.18 ± 0.39	0.130
Crude ash (%)	1.20 ± 0.07	1.13 ± 0.08	0.688

### 3.3. Dietary Green Alfalfa Supplementation Significantly Reduced Muscle Water Loss Rate and Increased Muscle Moisture Content in Diqing Tibetan Pigs

The meat quality and chemical composition results (Table 5) showed that, compared with the CON group, the GA group had a significantly reduced water loss rate, with a relative reduction of 17.80%, and a significantly increased muscle moisture content, with a relative increase of 3.51%.

### 3.4. Effects of Dietary Green Alfalfa Supplementation on Cecal Microbial Composition in Diqing Tibetan Pigs

**Figure 1:**
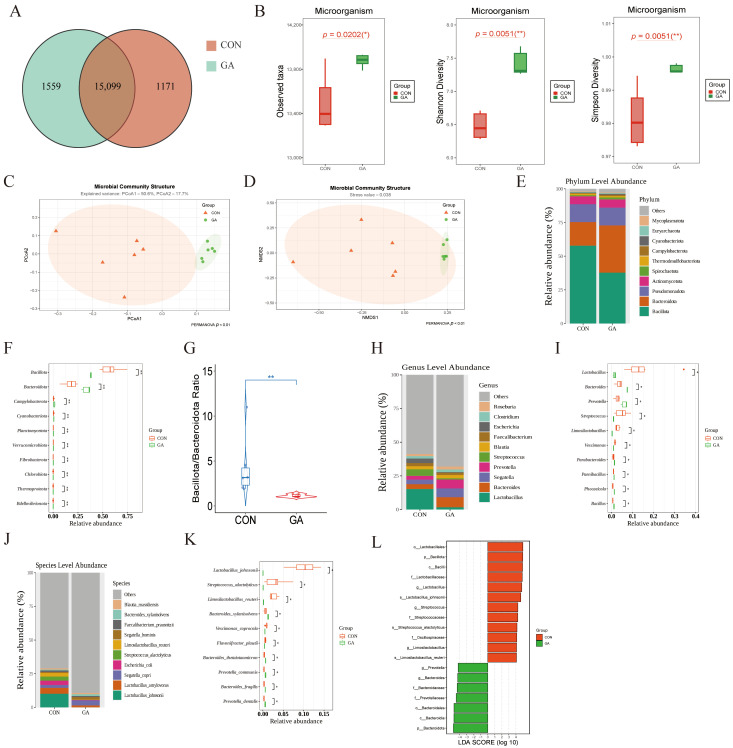
Effects of green alfalfa on cecal microbial composition and diversity in Diqing Tibetan pigs. (**A**) Venn diagram based on species-level detected taxa; (**B**) alpha-diversity analysis, including observed species-level taxa, the Shannon index, and the Simpson index; (**C**) principal coordinates analysis (PCoA); (**D**) non-metric multidimensional scaling (NMDS); (**E**) phylum-level microbiota composition; (**F**) phylum-level differential analysis; (**G**) abundance ratio of Bacillota to Bacteroidota (Bacillota/Bacteroidota ratio, F/B); (**H**) genus-level microbial composition; (**I**) genus-level differential analysis; (**J**) species-level microbial composition; (**K**) species-level differential analysis; and (**L**) linear discriminant analysis (LDA) score plot from LEfSe analysis, showing only taxa with LDA scores ≥ 4.0. CON, basal diet control group; GA, green alfalfa-supplemented group. * and ** indicate *p* < 0.05 and *p* < 0.01, respectively. The isolated red point outside the whiskers in panel I represents an outlier in the CON group.

To investigate the effects of green alfalfa feeding on the gut microbiota of Diqing Tibetan pigs, cecal contents were subjected to metagenomic sequencing and analysis. A total of 17,829 detected species-level taxa were identified. The two groups shared 15,099 detected species-level taxa, whereas 1171 and 1559 taxa were specific to the CON and GA groups, respectively (Figure 1A). Green alfalfa supplementation significantly increased the alpha-diversity indices of observed taxa and the Shannon and Simpson indices (Figure 1B). PCoA and NMDS analyses of beta diversity revealed highly significant differences in microbial communities between the two groups (Figure 1C,D).

To further examine changes in microbial composition and abundance, the top 10 cecal microbial taxa by relative abundance at the phylum, genus, and species levels were analyzed. At the phylum level, Bacillota and Bacteroidota were the dominant phyla (Figure 1E). Compared with the CON group, the GA group showed a highly significant decrease in the abundance of Bacillota and a highly significant increase in the abundance of Bacteroidota (Figure 1F), resulting in a highly significant decrease in the Bacillota/Bacteroidota ratio (F/B ratio) (Figure 1G). At the genus level, *Lactobacillus*, *Bacteroides*, and *Prevotella* were the dominant genera (Figure 1H), and the abundances of *Bacteroides*, *Prevotella*, and *Parabacteroides* were significantly increased in the GA group (Figure 1I). At the species level, *Lactobacillus johnsonii* and *Lactobacillus amylovorus* were the dominant species (Figure 1J). The abundance of *Lactobacillus johnsonii* was significantly decreased, whereas the abundances of *Bacteroides xylanisolvens*, *Bacteroides thetaiotaomicron*, and *Bacteroides fragilis* were significantly increased in the GA group (Figure 1K). The LDA score plot from LEfSe analysis (Figure 1L) showed that Bacillota was significantly enriched in the CON group, whereas Bacteroidota and its genera *Bacteroides* and *Prevotella* were significantly enriched in the GA group.

### 3.5. Dietary Green Alfalfa Supplementation Increased the Abundance of Functional Genes Related to Lipid and Vitamin Metabolism in the Cecal Microbiota of Diqing Tibetan Pigs

**Figure 2:**
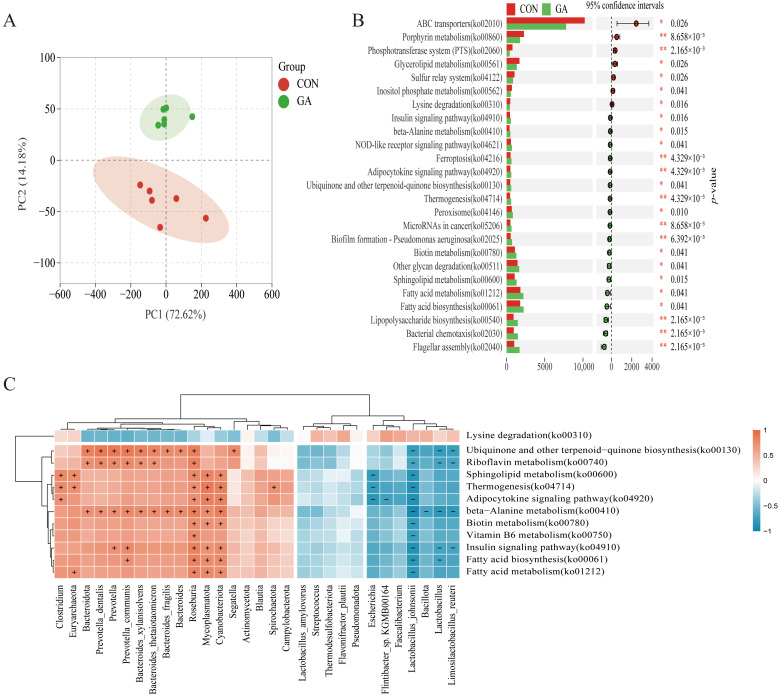
EggNOG-based functional prediction of cecal microbiota. (**A**) PCA; (**B**) differentially abundant functional pathways in the gut microbiome; (**C**) association analysis between microbial species in cecal contents and predicted functional pathways. * and ** indicate *p* < 0.05 and *p* < 0.01, respectively.

To further investigate the effects of green alfalfa on the functional gene composition and potential functions of cecal microorganisms, functional annotation was performed using the EggNOG database based on metagenomic assembly and gene prediction results, followed by mapping to relevant KEGG functional pathways. PCA showed that the two groups formed separate clusters (Figure 2A). Significance testing showed that, compared with the CON group, the GA group had significantly higher abundances of genes associated with KEGG functional pathways such as fatty acid metabolism (ko01212), sphingolipid metabolism (ko00600), adipocytokine signaling pathway (ko04920), vitamin B6 metabolism (ko00750), riboflavin metabolism (ko00740), and biotin metabolism (ko00780) (Figure 2B). To further identify microbial taxa associated with changes in predicted functional pathways related to lipid and vitamin metabolism, Pearson correlation analysis was used to calculate correlation coefficients between cecal microorganisms and predicted functional pathways. Ubiquinone and other terpenoid-quinone biosynthesis and β-alanine metabolism were positively correlated with Bacteroidota, *Bacteroides*, *Prevotella*, *Bacteroides thetaiotaomicron*, *Bacteroides xylanisolvens*, and *Bacteroides fragilis*. Riboflavin metabolism was positively correlated with Bacteroidota, *Prevotella*, *Bacteroides thetaiotaomicron*, and *Bacteroides xylanisolvens*. Ubiquinone and other terpenoid-quinone biosynthesis, riboflavin metabolism, sphingolipid metabolism, thermogenesis, adipocytokine signaling pathway, β-alanine metabolism, biotin metabolism, vitamin B6 metabolism, insulin signaling pathway, fatty acid biosynthesis, and fatty acid metabolism were negatively correlated with *Lactobacillus johnsonii* (Figure 2C).

### 3.6. Effects of Dietary Green Alfalfa Supplementation on the LD Transcriptomic Profile and Functional Pathways in Diqing Tibetan Pigs

**Figure 3:**
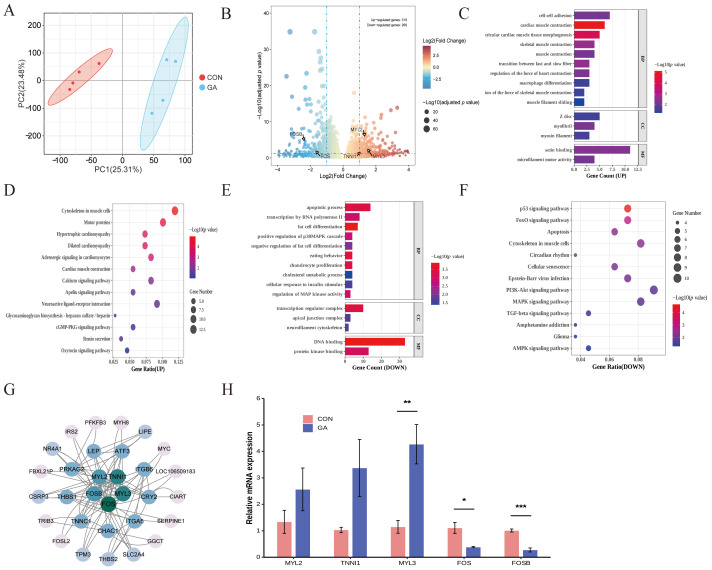
LD transcriptomic profile. (**A**) PCA. (**B**) Volcano plot. (**C**) GO enrichment analysis of upregulated DEGs. (**D**) KEGG enrichment analysis of upregulated DEGs. (**E**) GO enrichment analysis of downregulated DEGs. (**F**) KEGG enrichment analysis of downregulated DEGs. (**G**) PPI network of DEGs. (**H**) RT-qPCR analysis. *, ** and *** indicate *p* < 0.05, *p* < 0.01 and *p* < 0.001, respectively.

RNA-seq analysis of the LD muscle showed that samples clustered into two distinct groups (Figure 3A). A total of 587 differentially expressed genes (DEGs) were identified between the two groups, including 319 upregulated and 268 downregulated DEGs in the GA group (Figure 3B). The upregulated DEGs were significantly enriched in GO terms such as skeletal muscle contraction and transition between fast and slow fiber types (Figure 3C), as well as KEGG pathways such as Cytoskeleton in muscle cells and the Apelin signaling pathway (Figure 3D). The downregulated DEGs were significantly enriched in GO terms such as fat cell differentiation and regulation of MAP kinase activity (Figure 3E), as well as KEGG pathways such as the MAPK signaling pathway and FoxO signaling pathway (Figure 3F). The interaction network of significantly differentially expressed genes (Figure 3G) showed that Fos proto-oncogene (FOS), FBJ murine osteosarcoma viral oncogene homolog B (FOSB), slow skeletal troponin I (TNNI1), myosin light chain 2 (MYL2), and myosin light chain 3 (MYL3) had the highest degree values and were located at the core of the network. Compared with the CON group, the muscle growth and development related genes TNNI1, MYL2, and MYL3 were significantly upregulated, whereas the fat deposition related genes FOS and FOSB were significantly downregulated in the GA group (Figure 3B). RT-qPCR analysis of these five genes showed expression trends consistent with the RNA-seq results (Figure 3H).

### 3.7. Effects of Dietary Green Alfalfa Supplementation on the LD Metabolic Profile and Functional Pathways in Diqing Tibetan Pigs

**Figure 4:**
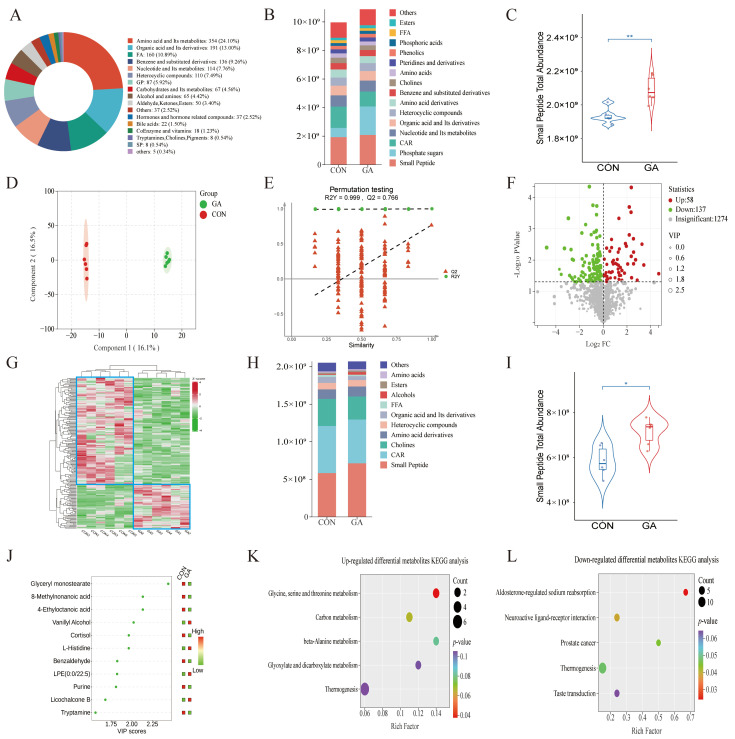
LD metabiolic profile. (**A**) Primary classification of identified metabolites. (**B**) Secondary classification of all identified metabolites. (**C**) Peptide content among identified metabolites. (**D**) OPLS-DA. (**E**) OPLS-DA permutation test plot (200 permutation tests). (**F**) Volcano plot of differential metabolites. (**G**) Heatmap of differential metabolites. (**H**) Secondary classification and composition of differential metabolites. (**I**) Significance analysis of oligopeptide content in differential metabolites. (**J**) Differential metabolites associated with meat quality. (**K**) KEGG analysis of upregulated differential metabolites. (**L**) KEGG analysis of downregulated differential metabolites. * and ** indicate *p* < 0.05 and *p* < 0.01, respectively.

Widely targeted metabolomics was used to investigate the effects of green alfalfa treatment on the metabolite composition of the LD muscle. A total of 1469 metabolites were identified across the two groups (Table S3). Based on metabolite features, the primary categories included amino acids and their metabolites (24.10%), organic acids and their derivatives (13.00%), fatty acyls (10.89%), benzene and substituted derivatives (9.26%), nucleotides and their metabolites (7.76%), heterocyclic compounds (7.49%), glycerophospholipids (5.92%), carbohydrates and their metabolites (4.56%), and coenzymes and vitamins (1.23%) (Figure 4A). The main secondary categories included small peptides, phosphate sugars, acylcarnitines, nucleotides and their metabolites, and organic acids and their derivatives (Figure 4B). Small-peptide content was significantly higher in the GA group than in the CON group (Figure 4C). OPLS-DA showed clear separation between the CON and GA groups, with good within-group repeatability (Figure 4D). The original OPLS-DA model had a fitting parameter R^2^Y = 0.999 and a cross-validated predictive parameter Q^2^ = 0.766. The model was further validated using 200 permutation tests, which showed that the intercept of the Q^2^ regression line on the y-axis was less than 0 and that all Q^2^ values of the permuted models were lower than that of the original model, indicating good cross-validated predictive ability of the OPLS-DA model (Figure 4E).

Using *p* < 0.05 and VIP > 1.0 as thresholds, 195 differential metabolites (DEMs) were identified, including 58 upregulated and 137 downregulated metabolites (Figure 4F). Clustering analysis showed that the DEMs formed two distinct clusters, with clear differences between the two groups (Figure 4G). Based on the features of the DEMs, the differential metabolites included small peptides, acylcarnitines, cholines, amino acid derivatives, heterocyclic compounds, organic acids and their derivatives, free fatty acids, and alcohols (Figure 4H). Small peptide content was significantly increased in the GA group (Figure 4I). The contents of vanillyl alcohol, L-histidine, licochalcone B, and LPE (0:0/22:5) were significantly increased, whereas the contents of glyceryl monostearate, benzaldehyde, cortisol, tryptamine, 4-ethyloctanoic acid, 8-methylnonanoic acid, and purine were significantly decreased (Figure 4J). KEGG enrichment analysis of DEMs showed that upregulated DEMs were significantly enriched in the glycine, serine, and threonine metabolism pathway (Figure 4K), whereas downregulated DEMs were significantly enriched in the thermogenesis pathway (Figure 4L).

### 3.8. Integrated Analysis of Cecal Metagenomic, LD Transcriptomic, Metabolomic, and Muscle Phenotypic Data

**Figure 5:**
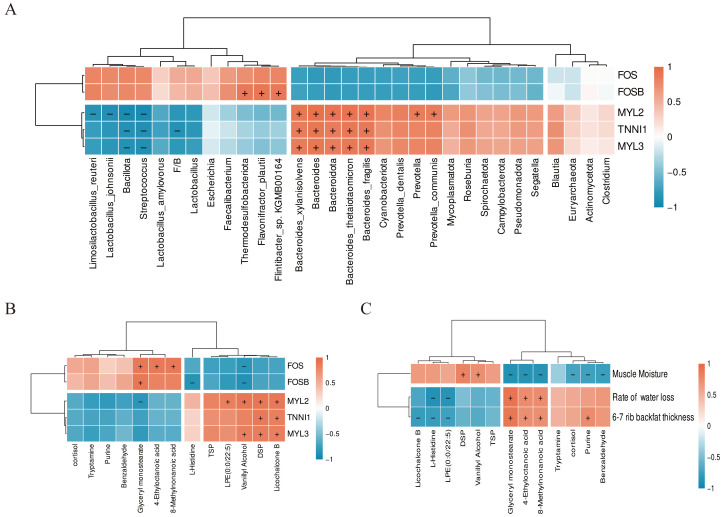
Integrated analysis of the metagenome of cecal contents, LD transcriptome, metabolome, and meat quality. (**A**) Association results between the metagenome of cecal contents and the LD transcriptome. (**B**) Association results between the LD transcriptome and the metabolome. (**C**) Association results between the LD metabolome and meat quality (TSP: total content of small-peptides among all detected metabolites; DSP: total content of small peptides among identified differential metabolites).

Association analyses among the cecal microbiota, LD transcriptome, LD metabolome, and muscle phenotypes were performed to explore the potential mechanisms underlying the effects of green alfalfa treatment on meat quality in Diqing Tibetan pigs (Table S4). The association analysis between the cecal microbiota and transcriptome (Figure 5A) showed that Bacteroidota, *Bacteroides*, *Bacteroides thetaiotaomicron*, *Bacteroides xylanisolvens*, and *Bacteroides fragilis* were positively correlated with *MYL*2, *TNNI*1, and *MYL*3; the F/B ratio was negatively correlated with *TNNI*1; Bacillota was negatively correlated with *MYL*2, *TNNI*1, and *MYL*3; and *Lactobacillus johnsonii* was negatively correlated with *MYL*2. The transcriptome-metabolome association analysis (Figure 5B) showed that *MYL*2 was positively correlated with licochalcone B, DSP, vanillyl alcohol, and LPE (0:0/22:5), and negatively correlated with glyceryl monostearate; *TNNI*1 was positively correlated with licochalcone B and DSP; *MYL*3 was positively correlated with licochalcone B, DSP, and vanillyl alcohol; *FOS* was positively correlated with 4-ethyloctanoic acid, 8-methylnonanoic acid, and glyceryl monostearate, and negatively correlated with vanillyl alcohol; and *FOSB* was positively correlated with glyceryl monostearate and negatively correlated with L-histidine and vanillyl alcohol. The association analysis between the metabolome and muscle phenotypes (Figure 5C) showed that purine, 4-ethyloctanoic acid, 8-methylnonanoic acid, and glyceryl monostearate were positively correlated with 6–7 rib backfat thickness; 4-ethyloctanoic acid, 8-methylnonanoic acid, and glyceryl monostearate were positively correlated with water loss rate; DSP and vanillyl alcohol were positively correlated with muscle moisture content; licochalcone B, L-histidine, and LPE (0:0/22:5) were negatively correlated with 6–7 rib backfat thickness; L-histidine and LPE (0:0/22:5) were negatively correlated with water loss rate; and glyceryl monostearate, 4-ethyloctanoic acid, 8-methylnonanoic acid, cortisol, purine, and benzaldehyde were negatively correlated with muscle moisture content.

## 4. Discussion

Dietary fiber is an important component of pig diets, and appropriate fiber levels have positive effects on intestinal health and animal welfare. Although fiber has low digestibility and energy contribution and can negatively affect growth performance [23], Diqing Tibetan pigs have long inhabited plateau regions under mainly free-range conditions and have developed strong tolerance to roughage through natural selection. Their ability to degrade neutral detergent fiber (NDF) is significantly higher than that of DLY pigs [24]; their gut microbiota has high potential for fiber utilization; and they can maintain growth in closed production systems with diets containing 90% roughage [25]. In the present study, supplementation of Diqing Tibetan pig diets with 10% green alfalfa had no significant effects on final body weight, average daily gain, total dry matter intake, and dry matter feed conversion ratio, which is consistent with the findings of Li et al. [7] and Xu et al. [9]. The GA group showed a decreasing trend in average daily gain, but the between-group difference did not reach significance. The absence of significant differences in total dry matter intake and dry matter feed conversion ratio between the two groups indicates that 10% green alfalfa supplementation did not significantly affect the growth performance of Diqing Tibetan pigs.

The degree of obesity in pigs has a substantial impact on meat quality and also affects feed efficiency, reproductive performance, and health status [26]. Excessive fat deposition that results in a high fat percentage reduces consumer acceptance [27]. Diqing Tibetan pigs have a strong capacity for fat deposition, thick backfat, and a low lean meat percentage [18]. However, the present study showed that dietary green alfalfa supplementation significantly reduced 6–7 rib backfat thickness in Diqing Tibetan pigs. Average backfat thickness, fat percentage, and intramuscular fat tended to decrease, whereas loin eye area and lean meat percentage tended to increase, indicating that green alfalfa may improve fat deposition characteristics and enhance the potential for optimizing carcass composition in Diqing Tibetan pigs. Alfalfa is rich in dietary fiber, vitamins, saponins, polysaccharides, flavonoids, and other nutrients [6]. The reduction in backfat thickness caused by green alfalfa supplementation may be attributable to its dietary fiber, vitamins, saponins, polysaccharides, flavonoids, and other components. Dietary fiber can effectively reduce lipid accumulation in the liver and adipose tissue [28]; vitamins can regulate lipid metabolism through adipocyte differentiation, lipid synthesis and oxidation, insulin sensitivity, and related processes [29]; saponins can lower cholesterol and blood lipid levels [30]; polysaccharides can reshape gut microbial composition, promote the growth of beneficial bacteria, and indirectly influence lipid metabolism [31]; and flavonoids can regulate lipid metabolism by inhibiting adipogenesis and promoting lipolysis [32]. Therefore, under conditions of similar total dry matter intake and dry matter feed conversion ratio between the two groups, the significant reduction in 6–7 rib backfat thickness in the GA group may be related to the higher dietary fiber level in the GA diet and the combined regulation of lipid metabolism and inhibition of fat deposition by vitamins, saponins, polysaccharides, and flavonoids derived from green alfalfa.

With socioeconomic development and rising living standards, high-quality pork is increasingly favored by consumers [2]. Meat quality is mainly evaluated through physicochemical properties, sensory quality, and nutritional value, among which intramuscular fat (IMF) content, water-holding capacity, and tenderness are key indicators [33]. The results of this study indicated that crude protein and intramuscular fat contents in the GA group showed decreasing trends, which may affect the nutritional value and palatability of the meat. Low water-holding capacity leads to excessive fluid loss during cooking, thereby reducing tenderness and juiciness [34]. Feed composition has a marked effect on meat quality [35]. The addition of 10% alfalfa silage to DLY pig diets significantly reduced drip loss [9], which is generally consistent with the present findings. These results indicate that dietary green alfalfa supplementation significantly reduces water loss rate and increases muscle moisture content, thereby improving water-holding capacity and potentially improving consumers’ perception of pork juiciness. Green alfalfa supplementation in Tibetan pig diets may be suitable for free-range grazing and small-scale ecological pig production systems. Without impairing growth performance, it can reduce feeding costs and improve carcass traits and meat quality.

Pigs cannot secrete enzymes capable of digesting fiber and therefore rely on microorganisms in the large intestine for fiber degradation [36]. In Diqing Tibetan pigs, cecal microbial diversity and complexity are higher than those in the colon, and the cecum has greater fiber utilization capacity [25]. Metagenomic sequencing and analysis of cecal contents in the present study showed that dietary green alfalfa supplementation significantly increased the gut microbial alpha-diversity indices of observed taxa and the Shannon and Simpson indices. In the GA group, the abundances of Bacteroidota, *Bacteroides*, *Prevotella*, *Parabacteroides*, *Bacteroides thetaiotaomicron*, *Bacteroides xylanisolvens*, and *Bacteroides fragilis* were significantly increased, whereas the abundances of Bacillota and *Lactobacillus johnsonii* and the Bacillota/Bacteroidota ratio (F/B ratio) were significantly reduced. These results indicate that green alfalfa significantly altered the abundance of microbial taxa associated with fat deposition in Diqing Tibetan pigs. The green alfalfa used in this study contained 26.26% crude fiber, and increasing dietary fiber content can enhance microbial diversity [25]. Gut microbial diversity affects livestock and poultry production performance [37]. Fiber-based dietary interventions, including pectin [38], cocoa husk [39], and wheat bran [40], significantly decreased the abundance of Bacillota and increased the abundance of Bacteroidota in pigs, which is consistent with our findings. Bacillota directly regulates blood lipid levels [41] and is more abundant in obese individuals [42]. A higher F/B ratio is associated with stronger fat absorption and storage capacity [43]. *Bacteroides* can improve lipid metabolism [44] and is negatively correlated with body fat percentage and obesity [45]. Dietary fiber promotes *Prevotella* colonization in the intestine, improves glucose metabolism, promotes glycogen storage, and reduces the conversion of free glucose into fat, thereby reducing fat deposition [46]. *Parabacteroides* has been identified as a producer of secondary bile acids and succinate [47]. Bile acids activate the *TGR*5 receptor and increase the availability of free fatty acids through lipolysis, thereby promoting β-oxidation and thermogenic activity and reducing obesity [48]. *Bacteroides thetaiotaomicron* can promote enterohepatic folate metabolism, reduce hepatic monounsaturated fatty acids, increase polyunsaturated fatty acids, improve blood lipid profiles and insulin resistance, protect the liver [49], reduce plasma glutamate concentrations, and alleviate fat accumulation [45]. *Bacteroides xylanisolvens* enhances one-carbon metabolism by promoting folate synthesis, thereby enhancing lipid oxidation and reducing adipogenesis [50]. *Bacteroides fragilis* alleviates obesity through secondary metabolite production and is more abundant in groups with low abdominal fat [51]. *Lactobacillus johnsonii* promotes lipid synthesis-related gene expression and lipid deposition by regulating the gut microbiota [52]. In the present study, the abundances of microbial taxa associated with lipid metabolism increased, whereas those of taxa promoting fat deposition decreased, resulting in reduced adipogenesis.

Functional prediction of the gut microbiota showed that the GA group had significantly higher abundances of genes associated with pathways including vitamin B6 metabolism, riboflavin metabolism, biotin metabolism, ubiquinone and other terpenoid-quinone biosynthesis, β-alanine metabolism, fatty acid biosynthesis, fatty acid metabolism, and adipocytokine signaling. Vitamin B6 metabolism helps prevent obesity by optimizing amino acid and energy metabolism, glucose homeostasis, and hepatic lipid metabolism [53]. Riboflavin plays an important role in fatty acid β-oxidation [54]; riboflavin supplementation can activate fatty acid β-oxidation, the tricarboxylic acid cycle, and oxidative phosphorylation, thereby enhancing cellular metabolism [55]. Riboflavin deficiency upregulates fatty acid synthase and downregulates adipose triglyceride lipase, leading to hepatic lipid accumulation [56]. Biotin metabolism affects biotin synthesis and transport. Biotin is an essential cofactor for acetyl-CoA carboxylase, pyruvate carboxylase, propionyl-CoA carboxylase, and other enzymes, and it directly participates in lipid regulation. Biotin levels are significantly reduced in obese individuals, and biotin deficiency aggravates intestinal and systemic inflammation and promotes insulin resistance and lipid accumulation [57]. Biotin supplementation promotes β-oxidation and reduces white adipose tissue [58]. Impaired ubiquinone synthesis results in excessive mitochondrial reactive oxygen species production, which can lead to insulin resistance and fat deposition [59]. Ubiquinone supplementation improves mitochondrial function, enhances overall energy metabolism, and reduces fat deposition [60]. β-Alanine can serve as a substrate for pantothenic acid production, and pantothenic acid is a key precursor of coenzyme A (CoA) biosynthesis. CoA participates in phospholipid synthesis, fatty acid synthesis and degradation, and the tricarboxylic acid cycle [61]. β-Alanine can alleviate fat accumulation by restoring sulfur-containing amino acid metabolism [62]. Therefore, enhancement of vitamin B6 metabolism, riboflavin metabolism, biotin metabolism, ubiquinone and other terpenoid-quinone biosynthesis, and β-alanine metabolism in the GA group may promote lipid metabolism and inhibit adipogenesis by optimizing amino acid metabolism and β-oxidation and participating in the synthesis of lipid metabolism-related enzymes. Based on the correlation analysis between cecal microbiota and predicted functional pathways, Bacteroidota, *Bacteroides*, *Prevotella*, *Bacteroides thetaiotaomicron*, *Bacteroides xylanisolvens*, and *Bacteroides fragilis* may influence lipid metabolism through ubiquinone and other terpenoid-quinone biosynthesis and β-alanine metabolism. Bacteroidota, *Prevotella*, *Bacteroides thetaiotaomicron*, and *Bacteroides xylanisolvens* may influence lipid metabolism through riboflavin metabolism, ultimately reducing fat deposition.

The PPI network of significantly differentially expressed genes in the LD transcriptome showed that *TNNI*1, *MYL*2, *MYL*3, *FOS*, and *FOSB* were located at the center of the network. *TNNI*1 is associated with muscle growth and development; its overexpression significantly enhances the expression of genes related to muscle development, whereas its silencing has inhibitory effects [63]. As a marker for evaluating meat quality, *TNNI*1 is negatively correlated with shear force and cooking loss [64]. *MYL*2 is a key gene regulating muscle development. When *MYL*2 expression is increased, the expression levels of muscle development-promoting genes such as *MYH*1, *MYH*2, and *MYH*4 are upregulated, whereas the expression of *MSTN*, which inhibits muscle growth, is significantly reduced [65]. *MYL*2 may be associated with meat quality traits such as meat color, pH, tenderness, and drip loss by affecting myofibrillar contraction and postmortem rigor processes [66]. *MYL*3 is a candidate gene for muscle fiber formation. It is mainly involved in muscle contraction and is classified within processes such as muscle fiber development, muscle system processes, and regulation of myoblast differentiation [67]. *MYL*3 expression is higher in groups with greater tenderness, lower shear force, and more favorable amino acid composition [68]. In the present study, *TNNI*1, *MYL*2, and *MYL*3 were highly expressed in the GA group, which may promote muscle development. *FOS* is a key gene involved in fat deposition [69]; it is highly expressed in obese individuals [70] and affects glycolysis and cholesterol synthesis [71]. *FOSB* is a key gene involved in lipid metabolism [72]; it promotes fat deposition, whereas *FOSB* knockdown reduces fat deposition [73]. In the present study, *FOS* and *FOSB*, which are associated with lipid deposition, were significantly downregulated in the GA group, thereby reducing fat deposition and potentially explaining the significant reduction in 6–7 rib backfat thickness in this group.

Muscle metabolomic results showed that licochalcone B, TSP, DSP, vanillyl alcohol, L-histidine, and LPE (0:0/22:5) were significantly increased in the GA group, whereas glyceryl monostearate, benzaldehyde, cortisol, tryptamine, 4-ethyloctanoic acid, 8-methylnonanoic acid, and purine were significantly decreased. Licochalcone B promotes the proliferation and differentiation of muscle satellite cells, inhibits myostatin expression, and increases muscle yield [74]. In the present study, licochalcone B was highly enriched in the GA group, and the muscle development-promoting genes *TNNI*1, *MYL*2, and *MYL*3 were positively correlated with licochalcone B. Their synergistic effects may promote muscle growth and development and may be one of the reasons for the increasing trend in lean meat percentage in the GA group. Small peptides play important roles in functional nutrition in foods and are often used as sweeteners, color stabilizers, and flavor enhancers; they can also affect the retention of water and oil in foods [75]. Vanillyl alcohol is a widely used flavoring compound with sweet and creamy aromas [76,77]. L-histidine is a basic amino acid and a precursor of essential amino acids and bioactive dipeptides such as carnosine [78]. It can inhibit fat and protein oxidation, promote lipolysis, alter fatty acid composition, enhance the perceived intensity of salty and umami tastes [79], improve tenderness, increase water-holding capacity, and accelerate postmortem aging [80]. LPE (0:0/22:5) may positively affect the formation of flavor compounds in meat by interacting with microorganisms and promoting the formation and accumulation of volatile compounds associated with desirable meat flavor [81,82]. Excessive glyceryl monostearate can make meat more prone to oxidative spoilage, harden texture, reduce water-holding capacity, and impair meat color [83]. Benzaldehyde is a volatile compound with an unpleasant odor, and increased benzaldehyde content indicates lipid and protein oxidation [84]. As an indicator compound for quality deterioration in frozen red meat, benzaldehyde content is positively correlated with storage time [85]. Cortisol levels are significantly correlated with multiple meat quality indicators and can serve as an indicator for meat quality evaluation [86]. Reducing cortisol can improve meat quality [87]. Tryptamine levels are among the core indicators used to judge the freshness, safety, and edibility of meat [88], and tryptamine can produce off-odors that affect meat quality and pose potential health risks [89]. 4-Ethyloctanoic acid and 8-methylnonanoic acid are key contributors to “mutton-like” flavor and contribute distinctive off-flavors in meat [90]. Purine content is negatively correlated with tenderness, juiciness, oiliness, and overall sensory liking [91]. Overall, metabolites that promote muscle growth and development or have positive effects on flavor (TSP, DSP, vanillyl alcohol, L-histidine, and LPE (0:0/22:5)) were significantly upregulated in this study, whereas metabolites that adversely affect flavor and contribute to meat quality deterioration and oxidation (glyceryl monostearate, benzaldehyde, cortisol, tryptamine, 4-ethyloctanoic acid, 8-methylnonanoic acid, and purine) were significantly downregulated, thereby improving water-holding capacity and flavor.

**Figure 6:**
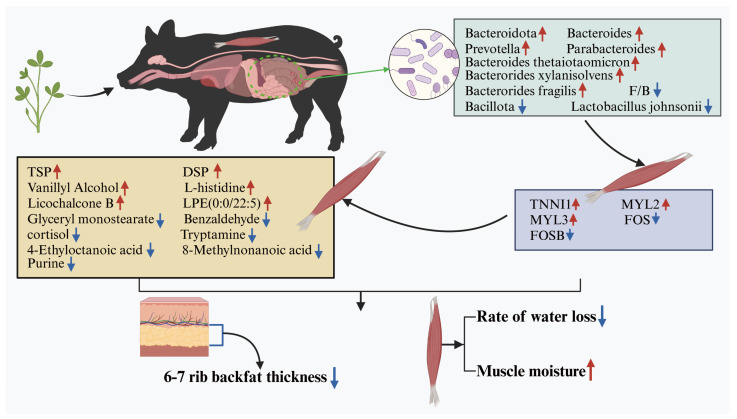
Hypothetical regulatory mechanisms by which dietary supplementation with green alfalfa regulates fat deposition and meat quality in Diqing Tibetan pigs (created with BioRender). The light green, light blue, and light yellow boxes represent the metagenomic, transcriptomic, and metabolomic results, respectively. Red upward and blue downward arrows indicate increases and decreases, respectively, whereas black arrows indicate the proposed relationships among the depicted components.

These results suggest a hypothetical regulatory mechanism by which dietary green alfalfa supplementation affects meat quality in Diqing Tibetan pigs: it increases the abundance of Bacteroidota, *Bacteroides*, *Prevotella*, *Parabacteroides*, *Bacteroides thetaiotaomicron*, *Bacteroides xylanisolvens*, and *Bacteroides fragilis*, which promote lipid metabolism, while decreasing the abundance of Bacillota and *Lactobacillus johnsonii*, which promote lipid deposition, and reducing the F/B ratio. Through the gut–muscle axis, the gut microbiota promotes the expression of muscle growth-related genes *TNNI*1, *MYL*2, and *MYL*3 and suppresses the expression of lipid deposition-related genes *FOS* and *FOSB*. It also increases the contents of licochalcone B, which promotes muscle growth and development, and vanillyl alcohol, L-histidine, and LPE (0:0/22:5), which positively affect flavor, while reducing the contents of glyceryl monostearate, benzaldehyde, cortisol, tryptamine, 4-ethyloctanoic acid, 8-methylnonanoic acid, and purine, which adversely affect flavor and contribute to meat quality deterioration and oxidation. Together, these effects significantly reduce 6–7 rib backfat thickness and water loss rate, significantly increase muscle moisture content, and improve muscle water-holding capacity in Diqing Tibetan pigs (Figure 6).

Moreover, this study has some limitations. These include the relatively small sample size, the use of a single breed, location, and GA inclusion level, the predictive nature of metagenomic functional annotation, the lack of sex × treatment interaction analysis, the hypothesis-generating nature of the correlation results, and the possible contribution of the lower digestible energy of the GA diet to the reduction in backfat thickness. Future studies should include additional breeds, larger sample sizes, and multiple study locations to further elucidate the causal relationships among green alfalfa, the gut microbiota, LD transcriptomic and metabolomic profiles, and production performance, thereby broadening the applicability of these findings.

## 5. Conclusions

Supplementation of Diqing Tibetan pig diets with 10% green alfalfa did not adversely affect growth performance and significantly reduced 6–7 rib backfat thickness and muscle water loss rate while increasing muscle moisture content. Green alfalfa reshaped cecal microbial composition, diversity, and function, as evidenced by increased alpha diversity (observed taxa, Shannon, and Simpson), increased abundances of lipid metabolism-promoting Bacteroidota, *Bacteroides*, *Prevotella*, *Parabacteroides*, *Bacteroides thetaiotaomicron*, *Bacteroides xylanisolvens*, and *Bacteroides fragilis*, and increased abundances of functional genes related to lipid and vitamin metabolism, along with decreased abundances of lipid deposition-promoting Bacillota and *Lactobacillus johnsonii* and a reduced F/B ratio. Green alfalfa increased the expression of muscle growth- and development-related genes *TNNI*1, *MYL*2, and *MYL*3 and decreased the expression of lipid deposition-promoting genes *FOS* and *FOSB*. It also increased the contents of licochalcone B, which promotes muscle growth and development, and vanillyl alcohol, L-histidine, and LPE (0:0/22:5), which positively affect flavor, while reducing the contents of glyceryl monostearate, benzaldehyde, cortisol, tryptamine, 4-ethyloctanoic acid, 8-methylnonanoic acid, and purine, which adversely affect flavor and contribute to meat quality deterioration and oxidation. Association analyses further showed significant relationships among gut microbiota, muscle transcriptome, metabolites, and meat quality traits. Specifically, significant associations were observed between microorganisms and genes, between genes and metabolites, and between metabolites and phenotypes. In summary, supplementation with 10% green alfalfa regulated gut microbial composition, diversity, and function; the expression of genes related to muscle growth, development, and lipid deposition; and metabolite composition, collectively affecting 6–7 rib backfat thickness and muscle water-holding capacity in Diqing Tibetan pigs. These findings provide a scientific basis for the direct utilization of green forage resources and offer a new perspective for mitigating competition between humans and livestock for grain and producing premium-quality pork.

## Figures and Tables

**Table 1:** Diet composition and nutrient levels (dry matter basis).

Diet Composition	CON	GA	Nutrient Level	CON (%)	GA (%)
Corn (%)	54.00	48.60	Digestible energy (MJ/kg)	12.65	12.26
Soybean meal (%)	11.10	9.99	Crude protein (%)	17.23	16.88
Rapeseed meal (%)	7.00	6.30	Crude fiber (%)	6.96	8.89
Wheat bran (%)	23.40	21.06	Crude fat (%)	3.66	3.71
Green alfalfa (%)	0.00	10.00	Lysine (%)	1.17	1.12
Limestone (%)	2.00	1.80	Calcium (%)	0.97	0.88
Salt (%)	0.55	0.50	Total phosphorus (%)	0.58	0.53
Premix (%)	1.95	1.75			

Note: The premix supplied the following per kg of diet: VA, 6500 IU; VD_3_, 2000 IU; VE, 42 IU; VK_3_, 2.00 mg; VB_1_, 2.00 mg; VB_2_, 6.40 mg; VB_6_, 3.00 mg; VB_12_, 25.00 μg; niacin, 1.20 mg; pantothenic acid, 20.00 mg; folic acid, 1.20 mg; biotin, 160.00 μg; nicotinamide, 25.00 mg; Fe (as ferrous sulfate), 100.00 mg; Cu (as copper sulfate), 25.00 mg; Mn (as manganese sulfate), 50.00 mg; Zn (as zinc sulfate), 80.00 mg; I (as potassium iodide), 500.00 μg; Se (as sodium selenite), 450.00 μg; methionine, 49.25 mg; L-lysine, 1443.00 mg; and choline chloride, 250.00 mg.

**Table 2:** Proximate nutrient composition and energy values of green alfalfa.

Moisture (%)	CP (%)	Crude Fat (%)	CF (%)	Ca (%)	P (%)	GE (MJ/kg)	DE (MJ/kg)
82.19 ± 0.96	13.73 ± 0.76	4.19 ± 0.12	26.26 ± 1.28	0.09 ± 0.02	0.04 ± 0.01	18.10	8.70

Note: Moisture is the measured value on a fresh weight basis. Crude protein, crude fat, crude fiber, calcium, and phosphorus are measured values on a dry matter basis. Gross energy (GE) and digestible energy (DE) for growing pigs are expressed on a dry matter basis; GE refers to the gross energy of green alfalfa itself, and DE refers to the digestible energy of green alfalfa in growing pigs.

## Data Availability

The data supporting the findings of this study have been deposited in the public repositories of the National Genomics Data Center (NGDC) of the China National Center for Bioinformation (CNCB). Specifically, the raw metagenomic sequencing data from cecal contents are available in the Genome Sequence Archive (GSA) under accession number CRA043207, and the transcriptomic sequencing data from longissimus dorsi muscle are available under accession number CRA043139. The metabolomics data from longissimus dorsi muscle are available in the OMIX database under accession number OMIX016980.

## Supplementary Materials

The following supporting information can be downloaded at https://www.mdpi.com/article/10.3390/foods15142528/s1. Table S1: Primer sequences used for RT-qPCR analysis; Table S2: Composition, concentrations, and supplier information of the internal standards used for metabolomic analysis; Table S3: All metabolites identified in the longissimus dorsi muscle; Table S4: Complete statistics for multi-omics correlation analyses.
