# Supplementary material for: Dietary Green Alfalfa Supplementation Reduces Backfat Thickness and Improves Muscle Water-Holding Capacity in Diqing Tibetan Pigs
Source: Foods. 2026 Jul 17;15(14):2528. doi: 10.3390/foods15142528 (PMC13409377; doi:10.3390/foods15142528)
Supplement: Supplementary file 1 [file foods-15-02528-s001.zip › Table S1. Primer sequences used for RT-qPCR analysis.pdf]

Table S1. Primer sequences used for RT-qPCR analysis.

Gene	Primer sequence (5′–3′)	Amplicon size (bp)
GAPDH	F: GACATCAAGAAGGTGGTGAAGCA<br>R: GTCGTACCAGGAAATGAGCTTGA	177
MYL2	F: GGGCTGATTATGTAAAGGA<br>R: CCGTGGGTGATGATGTG	142
TNNI1	F: CTGAAGCTCAAGGTGCTGGA<br>R: ACATGGCCTCGACGTTCTTT	217
MYL3	F: GCCTTCATGCTGTTTGACC<br>R: GTGCCCGTGTCCTTGTT	218
FOS	F: GATGTCTGTGGCTTCCCTTGA<br>R: TGCTGGGAACAGGAAGTCATC	184
FOSB	F: GCGTCTGGAGTTTGTGCTG<br>R: CGAGGAAGTGTACGAAGGGT	283
